# Hydrogen Sulfide Alleviates Alkaline Salt Stress by Regulating the Expression of MicroRNAs in *Malus hupehensis* Rehd. Roots

**DOI:** 10.3389/fpls.2021.663519

**Published:** 2021-07-26

**Authors:** Huan Li, Ting-Ting Yu, Yuan-Sheng Ning, Hao Li, Wei-Wei Zhang, Hong-Qiang Yang

**Affiliations:** State Key Laboratory of Crop Biology, College of Horticulture Science and Engineering, Shandong Agricultural University, Tai’an, China

**Keywords:** *Malus hupehensis* Rehd., microRNAs, hydrogen sulfide, alkaline salt stress, BGISEQ-500

## Abstract

*Malus hupehensis* Rehd. var. *pingyiensis* Jiang (Pingyi Tiancha, PYTC) is an excellent apple rootstock and ornamental tree, but its tolerance to salt stress is weak. Our previous study showed that hydrogen sulfide (H_2_S) could alleviate damage in *M. hupehensis* roots under alkaline salt stress. However, the molecular mechanism of H_2_S mitigation alkaline salt remains to be elucidated. MicroRNAs (miRNAs) play important regulatory roles in plant response to salt stress. Whether miRNAs are involved in the mitigation of alkaline salt stress mediated by H_2_S remains unclear. In the present study, through the expression analysis of miRNAs and target gene response to H_2_S and alkaline salt stress in *M. hupehensis* roots, 115 known miRNAs (belonging to 37 miRNA families) and 15 predicted novel miRNAs were identified. In addition, we identified and analyzed 175 miRNA target genes. We certified the expression levels of 15 miRNAs and nine corresponding target genes by real-time quantitative PCR (qRT-PCR). Interestingly, H_2_S pretreatment could specifically induce the downregulation of mhp-miR408a expression, and upregulated mhp-miR477a and mhp-miR827. Moreover, root architecture was improved by regulating the expression of mhp-miR159c and mhp-miR169 and their target genes. These results suggest that the miRNA-mediated regulatory network participates in the process of H_2_S-mitigated alkaline salt stress in *M. hupehensis* roots. This study provides a further understanding of miRNA regulation in the H_2_S mitigation of alkaline salt stress in *M. hupehensis* roots.

## Introduction

Salinity stress is an increasingly critical global agricultural problem. Salt affects plant root growth at the microcosmic level, reducing the quality and yield of crops (Munns and Tester, 2008; Zelm et al., 2020). The terrestrial soil affected by salt is mainly divided into three types: saline soil, alkaline soil, and saline alkali soil (Zhang et al., 2018). Neutral salt, NaCl, is the main component of salt stress, while NaHCO_3_ and Na_2_CO_3_ play a significant role in soil alkalization by increasing pH (Xu et al., 2019; Li et al., 2020). Under soil salinity and salinization, the primary stresses are osmotic stress and ionic toxicity in plants. These cause secondary damage, such as oxidative stress and nutrition disorders, that can damage plant cells, reduce production, and inhibit growth (Sun et al., 2015; Liang et al., 2018). The osmotic stress, ionic stress, and high pH caused by alkaline salt stress promote more direct toxicity than neutral salt stress (Shi and Wang, 2005; Yang et al., 2007). Except for plant halophytes adapted to salinization, most plants are salt sensitive. Therefore, optimizing crops in order to cultivate more salt-tolerant plants is an important strategy to improve crop yield and quality in salinized agricultural production (Zelm et al., 2020). Genetic engineering is a promising approach to improve plant salt tolerance, which is modulated by multiple genes and regulated at multiple levels. Deciphering the molecular genetic mechanisms associated with alkaline salt stress resistance can help researchers understand the complicated biological responses to alkaline salt and contribute to the genetic engineering of stress-resistant plants (Sun et al., 2015; Gao et al., 2016).

MicroRNAs (miRNAs) are evolutionarily conserved and single-stranded non-coding RNAs with a short length (about 21 nucleotides) that are major regulators of plant gene expression. miRNAs negatively modulate their targets by mRNA cleavage, DNA methylation, and the inhibition of translation. This process relies on a perfect complementary sequence between miRNA and target mRNA (Jones-Rhoades et al., 2006; Yu et al., 2017; Song et al., 2019). In addition to being a key regulator in plant development (D’Ario et al., 2017; Tang and Chu, 2017), numerous studies have reported that miRNAs are involved in the response of plants to various abiotic stresses, such as salinity (Zhu et al., 2013; Chen et al., 2015), alkalization (Cao et al., 2018; Xu et al., 2019), cold (Yang et al., 2013; Liu et al., 2017), drought (Li et al., 2008; Arshad et al., 2017), heat (Ding et al., 2017), and oxidative stress (Sunkar et al., 2006; Yue et al., 2017).

According to miRBase 22.1^1^, 10,405 miRNA sequences from 82 plants have been registered to date (Parmar et al., 2020). Some new and conserved miRNAs have recently been discussed in the salt responses of different plant species using next generation sequencing (NGS), which has greatly enriched the miRNA database. Forty-nine known (belonging to 28 miRNA families) and 22 predicted novel miRNAs were found to be differentially expressed in radish under salt stress (Sun et al., 2015). The most abundant miRNA families, MIR166, MIR156, and MIR171-1, may play critical roles during the response to salt in Paulownia (Fan et al., 2017). The overexpression of gma-miR172c, which was induced via abscisic acid (ABA), improved the tolerance of salinity and drought in *Arabidopsis* (Li et al., 2016). Like gma-miR862a, gma-miR5036, gma-miR1691-3p, and gma-398a/b were specifically induced by phosphorus deficiency and salinity stress in soybean root (Ning et al., 2019). MiRNVL5 and its target GhCHR were found in cotton, which can reduce Na^+^ uptake and enhance salt sensitivity as well as improve primary root growth and biomass in *A. thaliana* (Gao et al., 2016). A total of 75 differentially expressed miRNAs were identified and the expression of the miR390/tasiRNA-ARFs/ARF4 pathway was altered in cotton exposed to salinity (Yin et al., 2017). In Jerusalem artichoke, miR390 was induced by 100 mM NaCl and the miR390-TAS3-ARF model has an essential function in the regulation of Jerusalem artichoke in response to salt stress (Wen et al., 2020). However, reports on the comparative studies of miRNA expression profiles under alkaline stress are relatively scant. The miR156, miR159, miR398, miR319 (Zhu et al., 2011; Cao et al., 2018), miR139, miR172, miR408, miR169, and miR528 families (Xu et al., 2019) were observed to participate in salt or alkali stress responses in certain plant species.

Hydrogen sulfide (H_2_S), as well-known signal regulator, has been proposed to participate in various plant physiological processes and responses to abiotic stress. Evidence has proven that exogenous H_2_S can enhance salt tolerance in strawberry (Christou et al., 2013), *Medicago sativa* (Lai et al., 2014), rice (Mostofa et al., 2015), wheat (Deng et al., 2016), poplar (Zhao et al., 2018), and cucumber (Jiang et al., 2019).

Important breakthroughs in enhancing salt tolerance in plants using H_2_S have mainly focused on plant physiological and biochemical aspects, such as Na^+^/K^+^ balance, antioxidation system, photosynthesis, stomatal responses, and reactive oxygen species accumulation in the roots or leaves of plants. How H_2_S alleviates alkaline salt stress through miRNAs and the regulatory pathways of their target genes remains unclear and requires further study.

Apple trees are important economic fruit trees cultivated widely in temperate areas. In the apple industry, the salt in the soil is actually a mixture of NaCl, Na_2_SO_4_, NaHCO_3_, and Na_2_CO_3_ in addition to other salts. This mixture is alkaline, which is termed alkaline salt in soil science (Kawanabe and Zhu, 1991; Singh, 2019). Alkaline salt soil has seriously threatened the growth and production of crops including fruit trees such as apples. Cultivated apple plants are grafted from scions and rootstock. The salinization of soil is mainly caused by the stress of apple rootstock. *Malus hupehensis* Rehd. var. *pingyiensis* Jiang (Pingyi Tiancha, PYTC) is an excellent rootstock for apple cultivation due to its strong cold and water-logging resistance, but its tolerance to salt stress is weak (Yang et al., 2008; Zhang W. W. et al., 2019; Li et al., 2020). PYTC and *Malus domestica* Brokh belong to the same family and genus. Previous studies have shown that plant hormones (Wang X. X. et al., 2019), *MdMYB46* (Chen K. Q. et al., 2019), and *MdPUB29* (Han et al., 2019) play a vital regulatory role in improving the salt tolerance of apple. Moreover, some conserved miRNAs, such as miR396, 160, 393, miR159, 319, 164, and their targets were found to be responsible for bud growth and the formation of flower buds (Xing et al., 2016). mRNA and miRNA sequencing were used to understand the different flower-induced responses mediated by GA3 and its inhibitor paclobutrazol (PAC) (Fan et al., 2018). Zhang Y. et al. (2019) found that Md-miRln20 could suppress Md-TN1-GLS expression to negatively regulate Glomerella leaf spot (GLS) resistance in apple. At present, few reports have focused on the underlying regulation mechanism of miRNAs and their target genes in response to alkaline stress in *M. hupehensis.*

Our previous studies have shown that H_2_S pretreatment regulates oxidative stress and Na^+^/K^+^ homeostasis to mitigate alkaline salt stress in Malus hupehensis roots (Li et al., 2020). Here, we further investigated the molecular mechanism of H_2_S regulating tolerance to alkaline salt stress at the miRNA level through the analysis of differentially expressed miRNAs and their target genes in response to H_2_S and alkaline salt stress. We concluded that H_2_S alleviated alkaline salt stress due to several factors, including the specific induction of salt-tolerant miRNAs and improved elemental uptake, as well as changes in root architecture. Lastly, we established a comprehensive regulatory network based on miRNA-mediated H_2_S to mitigate alkaline salt stress responses in *M. hupehensis* roots.

## Materials and Methods

### Plant Culture and Different Treatments

This study was conducted at the National Research Center for Apple Engineering and Technology, Shandong Agriculture University (SDAU), Taian (36°18′ N, 117°13′ E), China. Seedlings of PYTC (*M. hupehensis*) were grown in black plastic bowls (diameter 11 cm, height 9 cm) that contained clean river sand, then cultivated in a greenhouse with a natural photoperiod. The seedlings were irrigated nutrition solution containing macronutrients and micronutrients every other day. When the seedlings reached the 6–7 leaf stage, they were pretreated with 0.5 mM sodium hydrosulfide (NaHS, H_2_S donor) for 72 h (changed every 24 h), which was dissolved in nutrient solution. Subsequently, they were transferred to nutrient solution for 1 day as an adaptation period. H_2_S-pretreated and alkaline salt stress treatments were carried out according to the method used by Li et al. (2020; Supplementary Figure 1).

Root samples were cut off carefully and rapidly frozen in liquid nitrogen at the end of the experiment, then stored at –80°C until extraction.

### Root sRNA Library Construction and Sequencing in BGISEQ-500 Platform

Total RNA was extracted from 0.1 g of frozen root samples using CTAB-PBIOZOL reagent (Hangzhou Bioer Technology Co. Ltd) according to the manufacturer’s instructions. Twelve small RNA libraries were constructed following the method of Zhu et al. (2018). Briefly, sRNAs were separated from the total RNA, and 18–30 nt (14–30 nt sRNA Ladder Marker, Takara) strips were selected and recycled. sRNAs were ligated using a 5′ and 3′ adaptor system. Subsequently, first strand reverse transcription was prepared and the cDNA fragments were enriched. In the purified PCR products, the final library was single strand circle DNA (ssCirDNA), which was then sequenced on a BGISEQ500 platform (BGI-Shenzhen, China).

### sRNA Annotation and miRNA Identification

We obtained clean reads by removing adaptors, 5′ primer contaminants, poly A tags, and small tags. We then summarized the length distribution of clean tags. Afterward, clean reads were mapped to reference genomes using other sRNA databases and Bowtie2^2^. For Rfam, we used cmsearch^3^. In the annotation information of different RNAs, to ensure that each small RNA was mapped to a unique category, we set the following rules: MiRbase > pirnabank > snoRNA(human/plant) > Rfam > other sRNA. Novel miRNA precursors were predicted by exploring the characteristic hairpin structure using miRA^4^.

### Differential Expression Analysis of miRNA

The RNA sequencing method was based on that of Jiang and Wong (2009). DEGseq (Wang et al., 2010) uses a binomial distribution statistical model to propose a new method according to the MA-plot (Yang et al., 2002). The calculated *P*-values of each gene are tested with multiple corrections by adjusting the *Q*-values (Benjamini and Hochberg, 1995; Storey and Tibshirani, 2003). We define the gene as a differentially expressed gene (DEG) when its ∣Fold Change∣ ≥ 2 and *Q*-value ≤ 0.001.

### Target Prediction of Known and Novel miRNAs

Various types of software were used to find more accurate targets. Target prediction of miRNAs was performed by psRobot or TargetFinder based on rules suggested by Fahlgren and Carrington (2010) and Wu et al. (2012).

### Functions of the Potential Targets of the Differentially Expressed miRNAs

To find significantly enriched differentially expressed target genes, all targets were mapped to the GO (gene ontology) terms database^5^ in order to find the genes that corresponded to specific biological functions. The discovery of significantly enriched GO terms was described using “GO:TermFinder^6^.”

The Bonferroni method was carried out to correct the *P*-value, and the threshold of *P*-value ≤ 0.05 (Abdi, 2007). The significantly enriched GO terms fulfilled the above conditions.

### Validation of miRNAs and Their Target Gene Expression by qRT-PCR

Total RNA was extracted from Control, H_2_S, H_2_S + AS, and AS treated roots according to the standard protocol of Trizol (Invitrogen, CA, United States). Total RNA was tailed addition reaction and first strand cDNA synthesis employed *TransScript*^®^ miRNA RT Enzyme Mix and 2 × TS miRNA Reaction Mix (TransGen Biotech, Beijing, China). The cDNA template for miRNA target gene qRT-PCR was synthesized using the TransScript^®^ All-in-One First-Strand cDNA Synthesis SuperMix for qPCR Kit (TransGen Biotech, Beijing, China). *PerfectStart*^TM^ Green qPCR Super Mix was used for qRT-PCR (TransGen Biotech, Beijing, China). Fifteen miRNAs and nine target genes were selected to conduct qRT-PCR and then verify the miRNA expression revealed by the RNA-seq. qRT-PCR was performed following the method used by Li et al. (2020). U6 and 18S rRNA were used as an internal standard to normalize the expression of miRNA and target genes. The roots from the Control treatment were taken as a reference sample and the relative expression level of genes was set to 1. All primers, including miRNAs and their targets in the qRT-PCR experiments, are shown in Supplementary Tables 3, 4.

### Statistical Analysis

Our experiments were constructed with 50 plants per treatment and three replications for each treatment. The least significant difference (LSD) test at the *P* < 0.05 level was used for the difference between treatments.

## Results

### Sequencing and Analysis of Twelve sRNA Libraries From *M. hupehensis* Roots

Twelve libraries were constructed from *M. hupehensis* roots subjected to pretreatment with H_2_S or alkaline salt. These libraries were sequenced by BGISEQ500 sequencing (Mortazavi et al., 2008; Wang et al., 2009), and the NCBI SRA database accession number was SRR13586165-SRR13586176. The raw reads generated from different libraries (Control, H_2_S, AS, and H_2_S + AS) ranged from 27.79 M to 30.05 M (Supplementary Table 1). The length distributions of cleaning tags obtained by filtering adapters, contaminants, and low-quality tags are shown in Supplementary Table 1. The length of sRNAs mainly ranged from 21 to 24 nt, and the 24 nt sRNAs manifested the most dominant length among the 12 libraries (Supplementary Figure 2), which was consistent with previous results in higher plants species, such as *M. hupehensis* and ‘Hanfu’ apple (Xing et al., 2014; Ma F. L. et al., 2020). Clean reads from Control, H_2_S, H_2_S + AS, and AS roots were mapped to the genome of domesticated apple in the Rfam database (see foot note 3) using cmsearch software. Furthermore, the clean reads were annotated into six different categories (Supplementary Table 2). Remarkably, a portion of unique sequences were unannotated sRNAs, which might be novel miRNAs in *M. hupehensis*.

### Identification of Known miRNA*s* in *M. hupehensis* Roots

**FIGURE 1:**
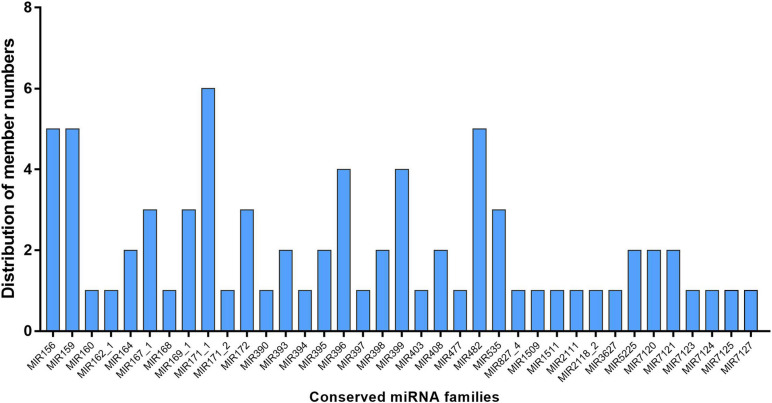
Distribution of conserved miRNA family members in *M. hupehensis* roots. 76 of 115 identified known miRNAs belong to 37 families.

**FIGURE 2:**
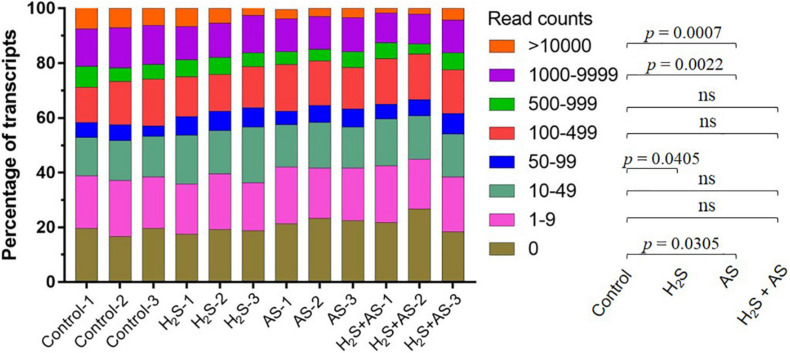
The read content frequencies of miRNAs in different libraries. Three biological repetitions were performed for each treatment. The significant differences (*P* < 0.05) between different treatment are shown on the right. AS, alkaline salt. ns, not significant.

To identify the known miRNAs in *M. hupehensis*, the sRNAs in the 12 libraries were aligned with the known miRNAs from *M. domestica* in the miRBase18.0 (see footnote 1) database using BLASTN. Only exactly matching sequences were considered. A total of 115 known miRNAs were identified, belonging to 37 miRNA families (Figure 1). Among them, the number of miRNA members in different families was roughly similar, with 1–3 members in most of the conserved miRNA families. Plenty of known miRNA families had many members, such as miR156 (six members), miR171_1 (seven members), miR482 (five members), and miR159 (six members). The mature miRNA sequences of identified known miRNAs are shown in Supplementary Table 5. In the 12 libraries, the known miRNA expression levels can also be expressed by the frequency of their reading content. According to the read count, the expression levels of known miRNAs in different treatments were divided into eight categories: 0 reads as no expression, 1–9 reads as lowest, 10–49 reads as lower, 50–99 reads as low, 100–499 reads as moderate, 500–999 reads as high, 1000–9999 reads as higher, and >10000 reads as highest (Xing et al., 2016). Our results suggested that the percentages of known miRNAs belonging to these eight categories in most libraries were extraordinarily similar (Figure 2). The largest percentage of known microRNA distributed in the no expression (0 reads) category ranged from 16.7 to 26.7% in different treatments, and the distribution in the highest (>10000 reads) category contained the lowest percentages in the six libraries at 1.7, 2.1, 4.2, 3.3, 2.9, and 3.3% in H_2_S + AS-1, H_2_S + AS-2, H_2_S + AS-3, AS-1, AS-2, and AS-3, respectively (Figure 2).

### Identification of Novel miRNAs in *M. hupehensis* Roots

Novel miRNA precursor was predicted by exploring the characteristic hairpin structure using miRA (see footnote 4). We identified a total of 15 novel miRNAs (for example, novel_mir10 and novel_mir11) from roots in different treatments. The mature sequence information of identified novel miRNAs is shown in Supplementary Table 6.

### Differentially Expressed miRNAs in *M. hupehensis* Roots

**FIGURE 3:**
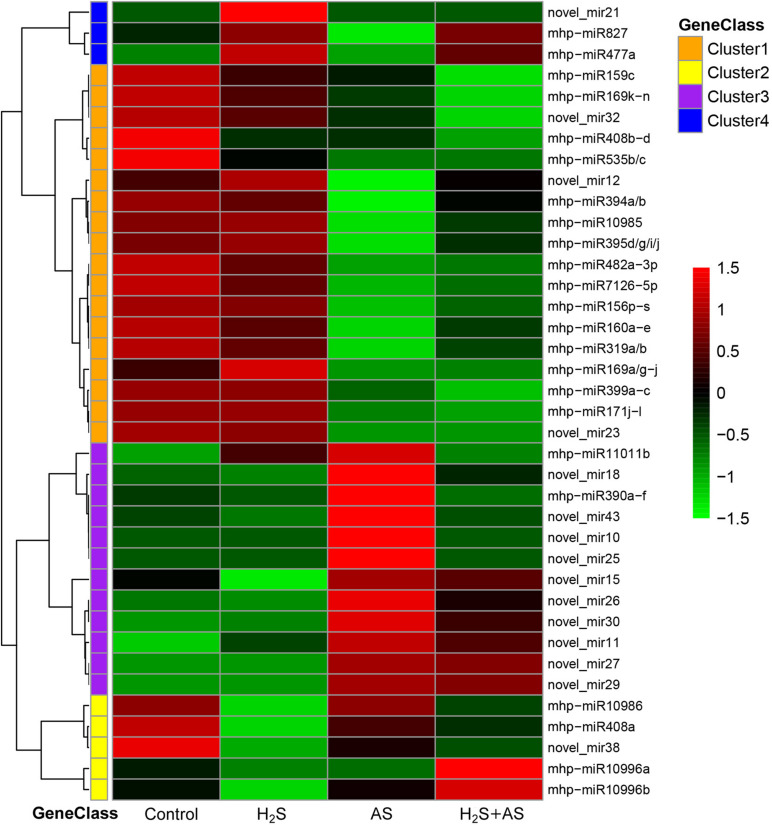
The expression levels of differential miRNAs identified in the roots of *M. hupehensis*. Differentially expressed miRNAs in apple under H_2_S, H_2_S + AS, and AS stresses as compared with control treatment. These miRNAs were clustered into four classes (Cluster 1–4).

**FIGURE 4:**
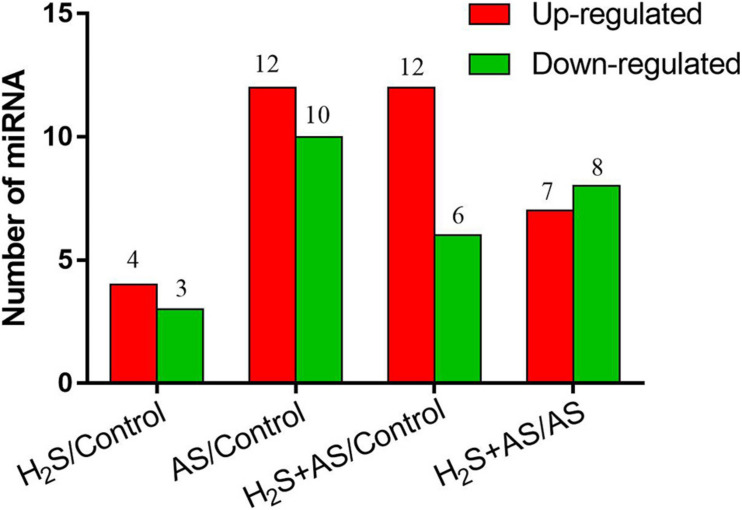
The number of upregulated and downregulated miRNAs. Number of differentially expressed miRNAs in *M. hupehensis* under H_2_S, H_2_S + AS, and AS stresses as compared with the control treatment.

To better identify the expression patterns of differentially expressed miRNAs in the Control, H_2_S, H_2_S + AS, and AS treatments, a statistical comparison of the miRNAs between them was performed. The expression levels of differentially expressed miRNAs were represented in a heatmap (Figure 3). We identified nine known and nine novel miRNAs as differentially expressed in H_2_S + AS compared with the control, including six downregulated and 12 upregulated genes (Table 1). In addition, the expressions of 12 miRNAs (2 known and 10 predicted novel) were upregulated, and 10 miRNAs (eight known and two predicted novel) were decreased by alkaline salt stress when compared with the control (Figure 4 and Table 1). Among the 15 miRNAs, seven were upregulated and eight were downregulated in H_2_S + AS compared with AS. Many alkaline salt-responsive miRNAs, such as mhp-miR394a, mhp-miR395d-5p, mhp-miR160a, and mhp-miR408, were markedly downregulated, suggesting that these miRNAs may be completely induced or inhibited by alkaline salt stress. Further analysis also found that the expression of mhp-miR477a and mhp-miR827 was induced by hydrogen sulfide. This further demonstrated the complexity of the miRNA regulatory mechanism of H_2_S mitigation of alkaline salt stress.

**TABLE 1:** Differentially expressed miRNAs in H_2_S alleviate alkaline salt and alkaline salt stress in *M. hupehensis* roots.

miR-name	Fold Change Log2 (H_2_S/Con)	miR-name	Fold Change Log2 (AS/Con)	miR-name	Fold Change Log2 (H_2_S + AS/Con)	miR-name	Fold Change Log2 (H_2_S + AS/AS)
mhp-miR408a	–1.84	mhp-miR11011b	1.13	mhp-miR11011b	1.09	mhp-miR319a/b-3p	1.15
mhp-miR408b-d	–1.06	mhp-miR156p-s	–1.08	mhp-miR10996a	1.67	mhp-miR10996a	1.14
mhp-miR827	5.78	mhp-miR160a-e	–2.77	mhp-miR160a-e	–1.10	mhp-miR160a-e	1.68
mhp-miR477a	1.64	mhp-miR319a/b-3p	–1.52	mhp-miR159c	–1.21	mhp-miR394a/b	2.64
mhp-miR169a/g-j	1.15	mhp-miR390a-f	1.03	mhp-miR10996b	1.54	mhp-miR477a	1.37
		mhp-miR394a/b	–3.20	mhp-miR169k-n	–1.03	mhp-miR827	6.09
		mhp-miR7126-5p	–1.23	mhp-miR399a-c	–1.11		
		mhp-miR482a-3p	–1.37	mhp-miR477a	1.97		
		mhp-miR535b/c	–1.09	mhp-miR827	5.81		
		mhp-miR395d/g/i/j	–3.81				

*AS, alkaline salt; Con, control treatment.*

### Prediction of Targets for Differentially Expressed miRNAs

In order to better understand the biological functions of the miRNA gene regulatory network of *M. hupehensis*, we used Targetfinder and psRobot to predict the target genes. Detailed annotation results of the target gene prediction are shown in Supplementary Tables 7, 8. In AS compared with control group, eight differentially expressed miRNAs targeted (six known and two novel miRNAs) 112 genes. Most of these miRNAs had multiple targets. Moreover, in H_2_S + AS compared with control group, the target genes for seven (six known and one novel miRNAs) differentially expressed miRNAs were predicted; these miRNAs targeted 100 genes. Six (four known and two novel miRNAs) differentially expressed miRNAs targeted 32 genes in H_2_S + AS compared with the AS group.

**FIGURE 5:**
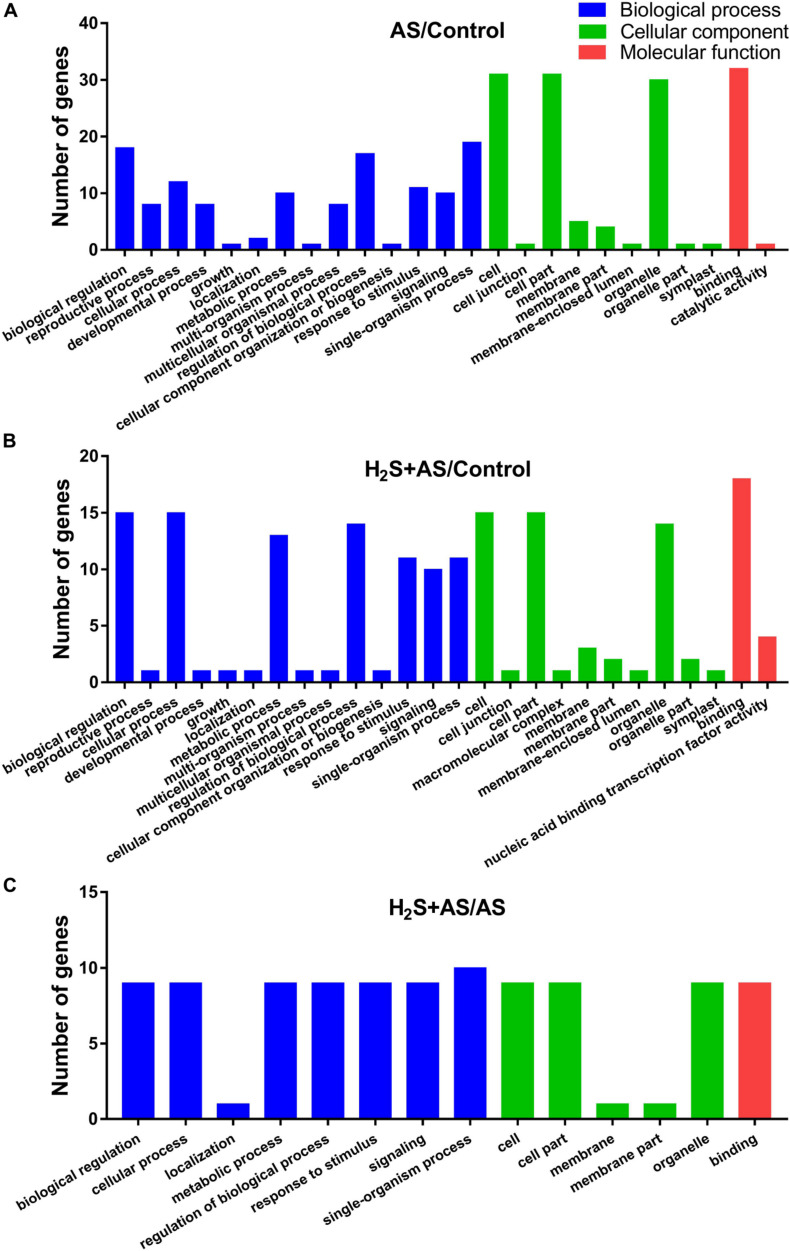
Gene ontology classification of predicted target genes for the identified miRNAs. Blue, green, and red represent three GO ontologies: biological process, cellular component, and molecular function, respectively. **(A)** Number of genes under AS stresses as compared with control treatment; **(B)** number of genes under H_2_S + AS stresses as compared with control treatment; **(C)** number of genes under H_2_S + AS stresses as compared with AS treatment.

**FIGURE 6:**
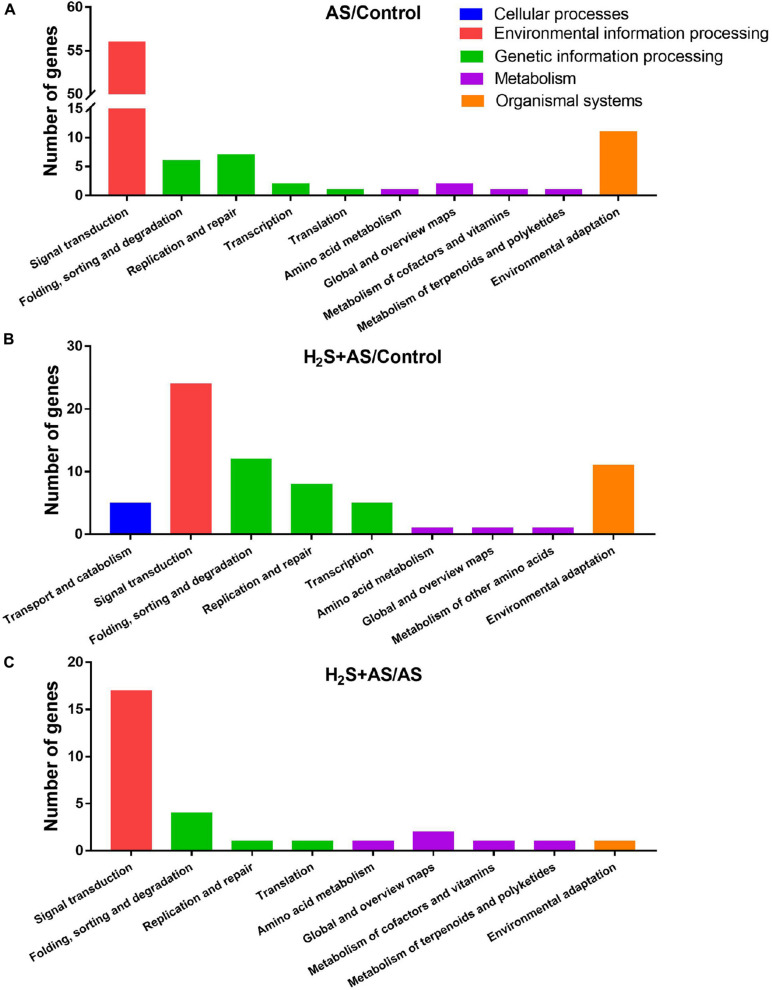
KEGG classification of predicted target genes for the identified miRNAs. Blue, red, green, purple, and orange represent five KEGG ontologies: Cellular processes, Environmental information processing, Genetic information processing, Metabolism, and Organismal systems, respectively. **(A)** Number of genes under AS stresses as compared with control treatment; **(B)** number of genes under H_2_S + AS stresses as compared with control treatment; **(C)** number of genes under H_2_S + AS stresses as compared with AS treatment.

GO and KEGG analyses were used to annotate the predicted target genes. GO analysis was carried out on the predicted targets based on cell composition, molecular function, and biological processes. GO analysis showed that a total of 100, 112, and 32 targets mainly participate in an extensive range of biological processes, including metabolic process, biological regulation, cellular process, regulation of biological process, multicellular organismal process, reproductive process, response to stimulus, signaling, single-organism process, and other biological processes in H_2_S + AS/Control, AS/Control, and H_2_S + AS/AS, respectively (Figures 5A–C). These targets also have multiple cellular components, such as cells, cell parts, membranes, membrane parts, and organelles (Figures 5A–C). As shown in Figure 5, the identified miRNA targets were involved in three molecular functions: binding, nucleic acid binding transcription factor activity, and catalytic activity. On the basis of the KEGG database, target genes were divided into five different processes: genetic information process, metabolism, tissue system, cell process, and environmental information process (Figures 6A–C). Many of these targets encoded stress-related transcription factors, such as auxin response factor (ARF) family, nuclear transcription factor Y, and WRKY transcription factor (Supplementary Tables 7, 8). Moreover, other predicted target genes encoding crucial proteins participated in diverse metabolic pathways, including TMV resistance protein, heat shock 70 kDa protein, SPX domain-containing membrane protein, and ribosomal RNA-processing protein.

### qRT-PCR Validation

**FIGURE 7:**
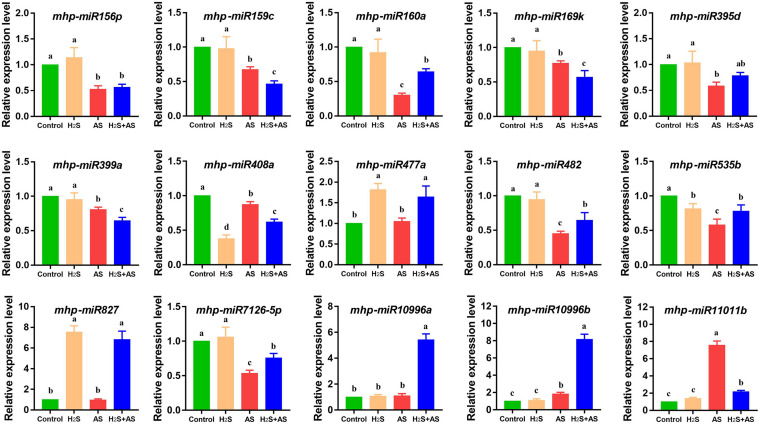
qRT-PCR analysis of identified miRNAs in *M. hupehensis* roots under different treatments. Three biological replicates were conducted for each sample and three technical replicates. The relative expression level of different miRNAs was calculated by the 2^–ΔΔ*CT*^ method. U6 was used for normalizing miRNA expression. The expression levels were normalized to those of the control. The standard error (SE) values are represented by the error bar, and the significant differences (*P* < 0.05) are indicated by different lowercase letters above each bar.

To validate the results of BGISEQ-500 sequencing and determine whether the dynamic expression observed in the H_2_S mitigation of alkaline salt stress was biologically consistent, qRT-PCR was used to analyze the expression of alkaline salt-response miRNAs. As expected, the data obtained showed that most detected miRNAs had a consistent expression change between sRNA sequencing and qRT-PCR. qRT-PCR analysis indicated that 14 of the 15 miRNAs were quantitatively consistent with the expression profile obtained by deep sequencing (Figure 7). Furthermore, we also investigated the correlation between miRNAs and their target genes in H_2_S pretreatment and alkaline salt stress. One downregulated and eight upregulated miRNAs among nine targeted genes were assayed by qRT-PCR. These results suggested that there was an approximate negative correlation between the expression of miRNAs and their corresponding targets.

## Discussion

Alkaline salt stress is a common factor in natural environments. The area affected by alkaline salt stress is increasing due to unreasonable irrigation, fertilization, and climate change. Long-term alkaline salt stress can inhibit plant growth and reduce crop yield, ultimately reducing the economic benefits of agricultural products and land use efficiency (Munns and Tester, 2008; Zelm et al., 2020). Some reports have determined that miRNAs are involved in the salt stress response of different plant species (Zhu et al., 2013; Chen et al., 2015). However, reports on the role of miRNAs in alkaline salt stress are limited. Apple is a major economic fruit that is widely planted in temperate regions worldwide (Boyer and Liu, 2004; Duan et al., 2017). Several studies have proven that exogenous H_2_S can relieve salt/alkali tolerance in many plant species (Christou et al., 2013; Mostofa et al., 2015; Zhao et al., 2018; Jiang et al., 2019; Li et al., 2020). Nevertheless, at present there is little information on the roles of H_2_S mitigation of alkaline salt regulation through miRNAs and their target genes in *M. hupehensis*.

In the present study, 115 known miRNAs and 15 novel candidate miRNAs were identified in *M. hupehensis*. The acquired sequences of these miRNAs were in accordance with the secondary structure criteria of miRNAs (Supplementary Tables 5, 6; Jiang et al., 2014; Hu et al., 2018). The length of *M. hupehensis* miRNAs in 12 libraries displayed more 24 nt than 21 nt miRNAs. sRNAs 24 nt in length were most abundantly expressed in libraries, suggesting that they could play a pivotal role in the response to alkaline salt stress. This is in agreement with previous studies in other plant species, such as *Arabidopsis*, *C. intermedia*, and bermudagrass (Rajagopalan et al., 2006; Zhu et al., 2013; Hu et al., 2018). The different sizes of sRNAs identified in this study had various functions in gene expression regulation. sRNAs may function as a broader modulation of gene expression during alkaline salt stress response.

Many miRNAs are highly conserved and widespread in plants, some of which are only observed-specific in plants (Jiang et al., 2014). The conserved miRNA family has only a few (0–2) nucleotide bases substituted in different plant species, and the abundance and number of the miRNA family members determines their diversity (Zhang et al., 2005; Sun et al., 2015). In the present research, conserved miRNA families had a relatively larger number of family members but a lower expression level when compared with non-conserved ones. The novel miRNAs represented non-conserved miRNA. Similarly, the known miRNAs represented conserved miRNAs, which could be divided into 37 miRNA families (Figure 1). The 115 known miRNAs belong to 37 conserved miRNA families, which are widely distributed in dicot and monocot model species (Jones-Rhoades et al., 2006; Rajagopalan et al., 2006; Lin et al., 2010). Some known miRNA families might have multiple members, such as miR156 (six members), miR171_1 (seven members), miR482 (five members), and miR159 (six members) (Figure 1). The results were consistent with those of Xing et al. (2016). Moreover, according to the read content’s frequencies, the expression levels of identified known miRNAs in the 12 libraries (Control, H_2_S, H_2_S + AS, and AS treatments) were subdivided into eight different categories, which were highly similar in the libraries (Figure 2). Our result indicated that these miRNAs participated in the regulation of H_2_S pretreatment induction in response to alkaline salt stress. Moreover, it was speculated that the differences in miRNAs’ reading number reflected their diverse expression levels in *M. hupehensis*. Many of the conserved miRNAs had higher read numbers. For example, mhp-miR398b/c, mhp-miR156, mhp-miR159d/e/f, and mhp-miR396a had extremely high read numbers, indicating that the expression of these miRNAs might be higher. Similar results have been reported in radish (*Raphanus sativus* L.) and bermudagrass (Sun et al., 2015; Hu et al., 2018).

A number of miRNAs have been observed to be salt/alkali stress regulated in diverse plant species, such as radish (Sun et al., 2015), *Zea mays* (Ding et al., 2009), Paulownia (Fan et al., 2017), *Medicago truncatula* (Cao et al., 2018), and tobacco (Xu et al., 2019). In our study, a total of 115 miRNAs were identified under H_2_S pretreatment and alkaline salt stress. However, several salt/alkali-responsive miRNAs, including miR159c, miR169, and miR399, did not show significant expression changes under alkaline salt stress in *M. hupehensis* roots. This contradiction implied that these miRNAs may be expressed in a plant-specific manner under alkaline salt stress. It was noteworthy that the expression of these salt/alkali-responsive miRNAs identified in our study might be fine-tuned in other species. For instance, miR156, miR169, and miR319 have been found to play important roles in flowering, heat, drought, cold, and salt stress responses (Sunkar et al., 2012; Wei et al., 2017; Cao et al., 2018; Song et al., 2019). This cross-kingdom regulation of gene expression indicates that these stress-related miRNAs could move from one species and target the mRNA of another interacting species in diverse stress responses (Song et al., 2019).

Here, 175 targets of 21 miRNAs were predicted (Supplementary Tables 7, 8). The GO analysis revealed that the dominant biological processes of these target genes were cellular process, biological regulation, metabolic process, regulation of biological process, reproductive process, and response to stimulus (Figure 5). This result indicates that these miRNA functions are correlated with enzyme metabolism, stress response, and growth transition in *M. hupehensis* under H_2_S pretreatment and alkaline salt treatment. Additionally, the pathway enrichment analysis of H_2_S pretreatment showed that these targets were involved in various pathways, including plant hormone signal transduction, DNA replication, MAPK signaling pathway–plant, plant–pathogen interaction, tryptophan metabolism, and glutathione metabolism (Figure 5), suggesting that these targets play critical roles in diversified biological processes by regulating the MAPK and hormone signal pathways involved in H_2_S alleviating alkaline salt stress.

Several stress response-specific miRNAs have been demonstrated in previous studies. Zhao et al. (2007) confirmed that the miRNA osa-miR169g was only induced by drought in rice. The specific up regulation of miRNA Athi-mir319c was caused by cold rather than salt, dehydration, or ABA stress (Sunkar and Zhu, 2004). In the present research, we compared the differentially expressed miRNAs in roots in response to H_2_S and alkaline salt stress. We found that H_2_S pretreatment induced specific upregulation of mhp-miR477a and mhp-miR827 and inhibited the expression of mhp-miR408. In addition, miR408 can regulate basic blue (Plantacyanin-like) protein in a very stable and conservative way (Maunoury and Vaucheret, 2011). A number of studies have shown that basic blue (plantacyanin) genes were increased during high salinity, heavy metal stress, oxidative stresses, and drought (Richards et al., 1998; Miller et al., 1999; Ma et al., 2011; Ruan et al., 2011). It has been confirmed that the overexpression of plantacyanin can promote cell growth at high salinity levels (Feng et al., 2013). The miR477 family is a miRNA family that can be induced by stress. Several studies have indicated that the miR477 family was differentially expressed during salt stress in maize, *Populus cathayana*, and *Salix matsudana* (Ding et al., 2009; Zhou et al., 2012). This result revealed that the mitigation effect of H_2_S on alkaline salt stress comprises the direct or indirect induction of more miRNAs and target genes resistant to alkaline salt stress. Based on this, we assume that H_2_S may have a unique induction mechanism on saline-tolerant miRNA and target genes, thus reducing the negative effects of alkaline salt stress on *M. hupehensis* roots. *M. hupehensis* is an excellent rootstock in the apple industry. However, it is sensitive to salt and alkali, which limits its application in saline-alkaline soil regions. In the future, it will be necessary to evaluate the miRNAs and target genes of H_2_S response to salt and alkali stress to understand the relationship between miRNAs and salt and alkali. miR827 and its target SPX have been identified to regulate the response to Pi starvation in rice (Aung et al., 2006; Lin et al., 2010). Consistent with miR827, miR399 and its target (ubiquitin-conjugating E2 enzyme, UBC) are involved in regulating phosphorus homeostasis (Chiou et al., 2006; Lin et al., 2010). In addition, we noticed that the expression of miR399 was downregulated by alkaline salt treatment. These findings seem to suggest that H_2_S may modulate the absorption of phosphorus and maintain phosphorus homeostasis, thus alleviating the damage caused by alkaline salt stress to *M. hupehensis* roots. However, the reasons behind this regulated phosphorus homeostasis pattern have not been determined. One of the challenges in the future is to completely decipher the molecular mechanism of phosphorus homeostasis in response to H_2_S and alkaline salt stress. Long-term salt stress can generally lead to secondary stress such as oxidative stress and nutritional imbalance in plants (Chinnusamy et al., 2006), and the increase of pH around the plant roots impedes the absorption of mineral nutrients (Capula-Rodríguez et al., 2016). Moreover, we found that there is a slight change in the pH of the growing medium before and after the irrigation with NaHS solution, all these pH values are suitable ranges for root growth.

In our study, the mhp-miR159c expression was decreased in response to H_2_S pretreatment and alkaline salt, which had more than one target. miR159-regulated MADS-box was also considered to modulate root development as well as regulating flowering time and salt stress (Saedler et al., 2001; Michaels et al., 2003; Guo et al., 2016). Meanwhile, *CaMADS* from pepper, as a positive stress response transcription factor, plays valuable role in the salt stress signaling pathway (Chen R. G. et al., 2019). In our study, the expressed miR159 and its target gene MADS-box JOINTLESS-like (MBP) may take part in H_2_S alleviating alkaline salt stress in the roots of *M. hupehensis*. Similar to MADS-box, miR169-targeted nuclear factor Y subunit A (NF-YA) in H_2_S pretreatment and alkaline salt treatment, which conditioned whole plant root architecture through altering specific cell type numbers and dimensions in the root meristem (Sorin et al., 2014), was widely regulated under salt and drought stress (Ni et al., 2013; Sun et al., 2015). Plant root architecture and morphological distribution directly affect the absorption of nutrients (including phosphorus) and water in soil, and further affect the growth and ecological function of the aboveground parts of plants (Lynch, 1995; Liao et al., 2001). The ideal root system configuration can guarantee the sustainable and stable development of forestry. In a previous study, we reported that H_2_S could alleviate the decrease in the numbers of absorbing roots and the inhibition of root activity under alkaline salt stress (Li et al., 2020). Taken together, these results reveal that miRNA mediated the improvement of root architecture, which may be the key to H_2_S alleviating alkaline salt stress. Therefore, it will be valuable to conduct an extensive evaluation of these miRNAs in the future to understand their potential in regulating root architecture.

**FIGURE 8:**
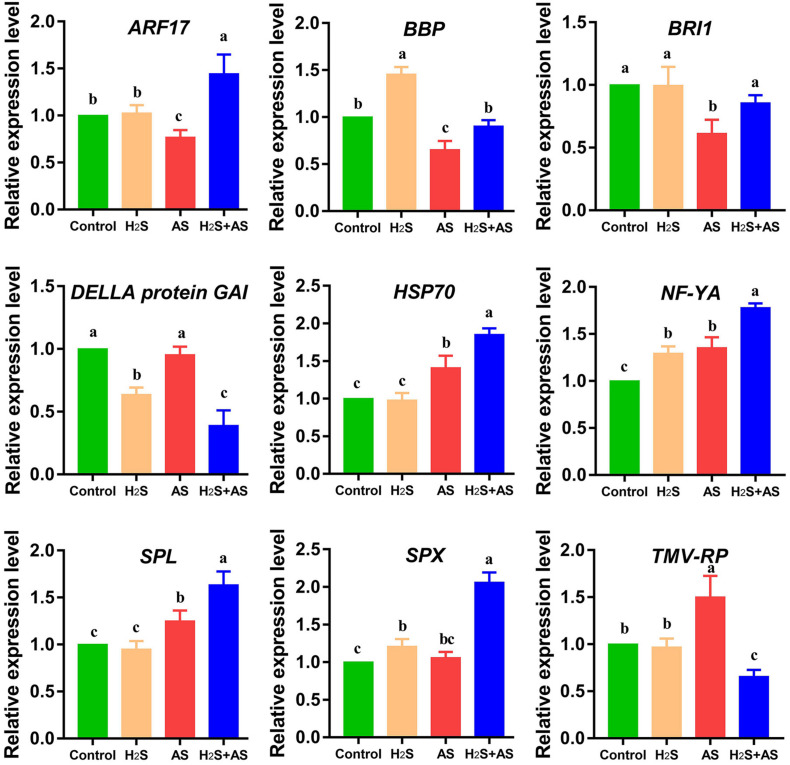
qRT-PCR analysis of predicted target genes of differentially expressed miRNAs in *M. hupehensis* roots under different treatments. Three biological replicates were conducted for each sample, and three technical replicates were conducted. The relative expression level of miRNAs was calculated by 2^–ΔΔ*CT*^ method. The 18S rRNA was used as internal reference for normalizing gene expression. The expression levels were normalized to those of the control. The standard error (SE) values are represented by the error bar, and the significant differences (*P* < 0.05) are indicated by different lowercase letters above each bar. ARF17, auxin response factor 17; BBP, basic blue protein-like; BRI1, receptor-like protein kinase BRI1-like 3; HSP70, heat shock 70 kDa protein; NF-YA, nuclear transcription factor Y subunit A-5-like; SPL, squamosa promoter-binding-like protein 18; SPX, SPX domain-containing membrane protein; TMV-RP, TMV resistance protein N-like.

In our results, alkaline salt decreased mhp-miR160a expression and increased mhp-miR160 target auxin response factors (ARF18) in *M. hupehensis* roots. Additionally, it was identified that miR160 participates in auxin signaling pathways and regulation of flower induction and growth (Sunkar et al., 2012; Xing et al., 2016). Similar results reported in salt-stressed *Populous tomentosa*, soybean, and radish that miR160 mediated target ARF regulation (Ren et al., 2013; Sun et al., 2015; Ning et al., 2019). Additionally, miR156p targeted SPL (SPL2/6/7), which was involved in conferring enhanced tolerance to Cd stress in *Arabidopsis* and regulating salt stress responses of *tamarisk* (Wang J. W. et al., 2019; Zhang et al., 2020). Moreover, the overexpression of MIR156a decreased salt resistance, while the overexpression of MdSPL13 targeting MdWRKY100 promoter strengthened salt tolerance in apple (Ma Y. et al., 2020). miR394 and its target F-box protein were reported to participate in the regulation of leaf inclination (Qu et al., 2019). The findings indicate that these miRNAs and their target genes might play prominent roles in *M. hupehensis* root adaptive response to alkaline salt stress, as an essential part of the alkaline salt stress regulation network in *M. hupehensis* (Figure 8).

## Conclusion

**FIGURE 9:**
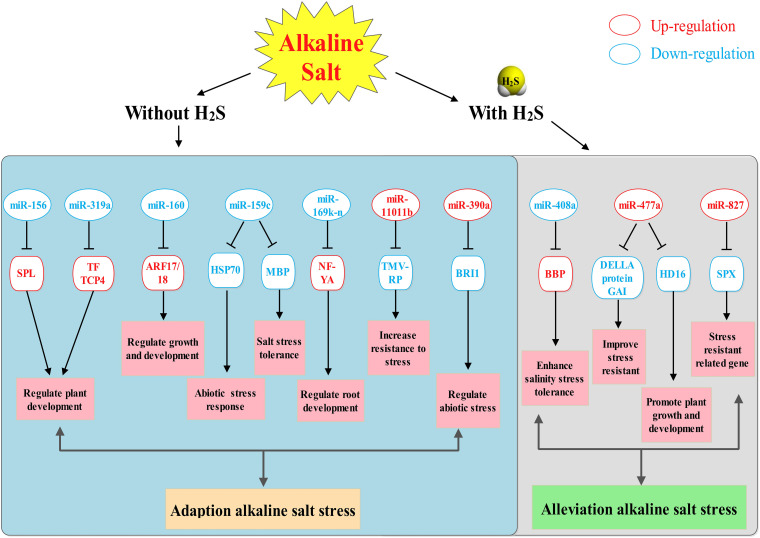
A potential regulatory network model of miRNAs in H_2_S alleviates alkaline salt stress in *M. hupehensis* roots. This model reveals how H_2_S alleviates the alkaline salt stress through mediating miRNAs. ARF17, auxin response factor 17; BBP, basic blue protein-like; BRI1, receptor-like protein kinase BRI1-like 3; HSP70, heat shock 70 kDa protein; NF-YA, nuclear transcription factor Y subunit A-5-like; SPL, squamosa promoter-binding-like protein 18; SPX, SPX domain-containing membrane protein; TMV-RP, TMV resistance protein N-like.

Our present study revealed that H_2_S could alleviate alkaline salt stress in *M. hupehensis* roots. Differentially expressed microRNAs and target genes involved in H_2_S and alkaline salt stress provide new insight for understanding the mechanism of how H_2_S alleviates alkaline salt stress in *M. hupehensis* roots. A total of 115 known miRNAs and 15 novel miRNAs were identified in alkaline salt treatment and H_2_S pretreatment. Furthermore, H_2_S specifically induces the expression of salt-tolerant mhp-miR408a and mhp-miR477a. In addition, the upregulated mhp-miR827 maintains phosphorus homeostasis in roots. Root architecture was improved by regulating the expression of mhp-miR159c and mhp-miR169 and their target genes. Collectively, these results suggest that H_2_S alleviates alkaline salt stress by contributing to the specific induction of salt-tolerant miRNAs and improved elemental uptake, as well as by inducing changes in root architecture (Figure 9). The current study revealed a miRNA-mediated network through which H_2_S alleviates alkaline salt stress in *M. hupehensis* roots.

## Data Availability Statement

The datasets presented in this study can be found in online repositories. The names of the repository/repositories and accession number(s) can be found in the article/Supplementary Material.

## Author Contributions

HuL prepared and wrote the manuscript. T-TY, Y-SN, and HaL performed the experiments and organized the methods and software. H-QY and W-WZ reviewed and edited the manuscript. All the authors read and approved the manuscript.

## Conflict of Interest

The authors declare that the research was conducted in the absence of any commercial or financial relationships that could be construed as a potential conflict of interest.

## Publisher’s Note

All claims expressed in this article are solely those of the authors and do not necessarily represent those of their affiliated organizations, or those of the publisher, the editors and the reviewers. Any product that may be evaluated in this article, or claim that may be made by its manufacturer, is not guaranteed or endorsed by the publisher.

## Footnotes

1 http://www.mirbase.org

2 http://bowtie-bio.sourceforge.net/bowtie2/index.shtml

3 http://www.sanger.ac.uk/resources/databases/rfam.html

4 https://github.com/mhuttner/miRA

5 http://www.geneontology.org/

6 http://www.yeastgenome.org/help/analyze/go-term-finder
