# Supplementary material for: Hydrogen Sulfide Alleviates Alkaline Salt Stress by Regulating the Expression of MicroRNAs in Malus hupehensis Rehd. Roots
Source: Front Plant Sci. 2021 Jul 26;12:663519. doi: 10.3389/fpls.2021.663519 (PMC8350742; doi:10.3389/fpls.2021.663519)
Supplement: Supplementary file 1 [file Data_Sheet_1.docx]

**Supplementary Material**

**Hydrogen sulfide alleviates alkaline salt stress by regulating the expression of microRNAs in *Malus hupehensis* Rehd. Roots**

*Huan Li^#^, Ting-ting Yu^#^, Yuan-sheng Ning, Hao Li, Wei-wei Zhang*, Hong-*

*qiang Yang**

College of Horticulture Science and Engineering, Shandong Agricultural University, State Key Laboratory of Crop Biology, 61 Daizong Street, Tai'an, Shandong 271018, PR China.

**Correspondence**

* Corresponding author,

*E-mail* *address*: zhangww@sdau.edu.cn

E-*mail* *address:* [hqyang@sdau.edu.cn](mailto:hqyang@sdau.edu.cn)

^#^ These authors contributed equally to this work.

**
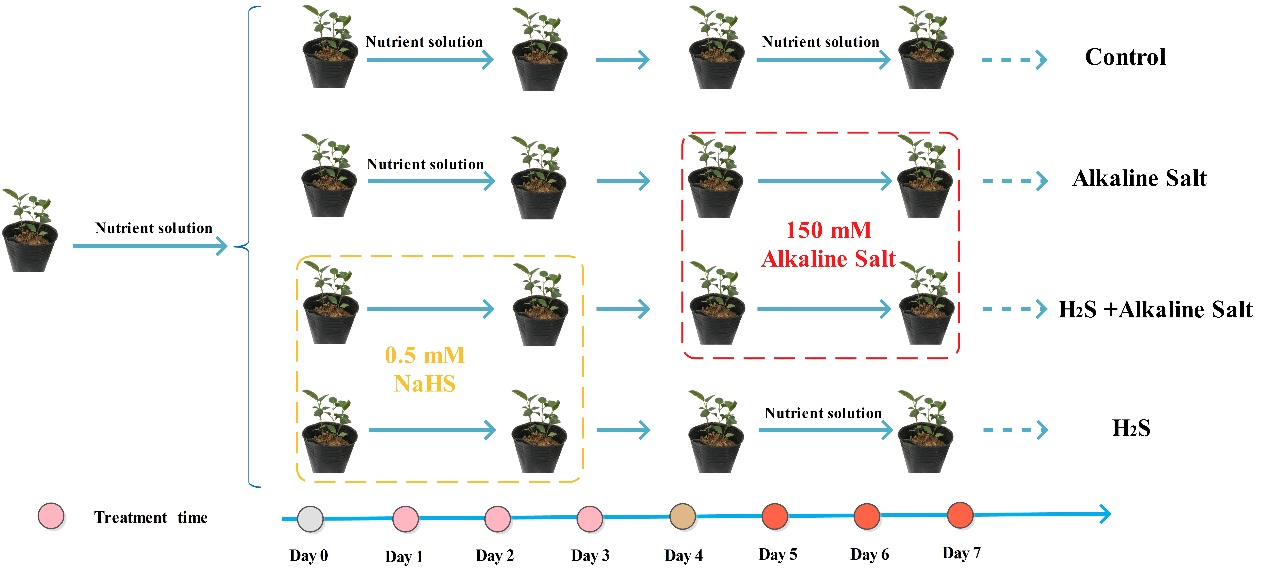
**

**Figure S1. Schematic diagram of different treatments in this study.**


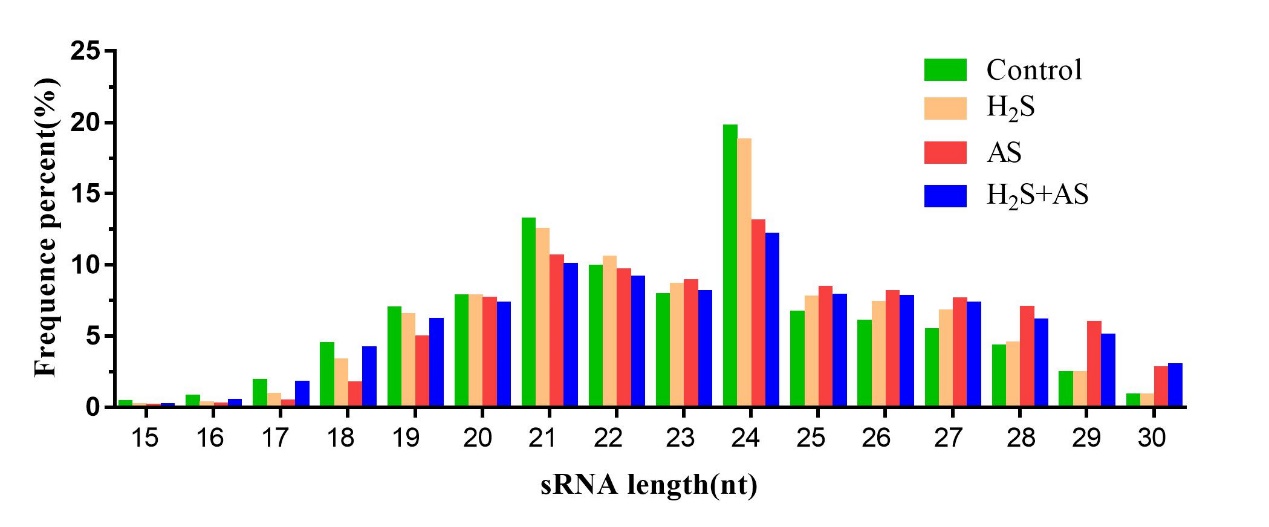


**Figure S2 Length distributions of sRNAs in different libraries in M. hupehensis roots.**

**Table S1. Statistics of clean reads mapping to genome in the twelve libraries of** ***M. hupehensis* roots.**

| Type | | Control | | H_2_S | | H_2_S+AS | | AS | |
| --- | --- | --- | --- | --- | --- | --- | --- | --- | --- |
|  |  | Total | % of raw reads | Total | % of raw reads | Total | % of raw reads | Total | % of raw reads |
| Raw reads | 1  2  3 | 28,108,187  29,661,652  28,330,313 | 100  100  100 | 27,783,113  28;963;869  27,949,209 | 100  100  100 | 29,012,269  28,973,126  28,497,863 | 100  100  100 | 30,046,957  29,000,514  28,199,591 | 100  100  100 |
| Clean reads | 1  2  3 | 25,052,010  27,137,651  25,925,308 | 89.13  91.49  91.51 | 25,860,576  27,193,125  25,853,019 | 93.08  93.89  92.50 | 26,211,637  27,587,699  26,423,897 | 90.35  95.22  92.72 | 28,237,326  27,483,623  26,482,981 | 93.98  94.77  93.91 |
| Mapped to genome | 1  2  3 | 14771068  15822120  14017337 | 58.96  58.30  54.06 | 13211990  16146243  14086277 | 51.09  59.37  54.49 | 17547416  16644375  15138712 | 66.94  60.33  57.29 | 22632624  21693154  20675244 | 80.16  78.93  78.07 |

**Table S2. Distribution of the small RNAs sequences in the twelve libraries of** ***M. hupehensis* roots.**

| Type | | Control | | H_2_S | | H_2_S+AS | | AS | |
| --- | --- | --- | --- | --- | --- | --- | --- | --- | --- |
|  |  | Total | % of raw reads | Total | % of raw reads | Total | % of raw reads | Total | % of raw reads |
| rRNA | 1  2  3 | 522118  542296  541395 | 2.08  2.05  2.09 | 558310  557121  445169 | 2.16  2.05  1.72 | 671928  1197848  774451 | 2.56  4.34  2.93 | 1025058  1225995  907234 | 3.63  4.46  3.43 |
| snoRNA | 1  2  3 | 6936  7579  5705 | 0.03  0.03  0.02 | 7212  7090  7182 | 0.03  0.03  0.03 | 10631  12903  10151 | 0.04  0.05  0.04 | 20246  20113  15405 | 0.07  0.07  0.06 |
| tRNA | 1  2  3 | 3523  2542  3974 | 0.01  0.01  0.02 | 4711  2939  3288 | 0.02  0.01  0.01 | 4755  9510  8282 | 0.02  0.03  0.03 | 10518  7649  8244 | 0.04  0.03  0.03 |
| snRNA | 1  2  3 | 2916  3656  3525 | 0.01  0.01  0.01 | 3115  3132  2398 | 0.01  0.01  0.01 | 3262  4960  4189 | 0.01  0.02  0.02 | 6228  6264  5133 | 0.02  0.02  0.02 |
| miRNA | 1  2  3 | 891394  792292  746423 | 3.56  2.97  2.88 | 592364  528653  654351 | 2.3  1.94  2.53 | 241319  249845  431465 | 0.92  0.92  1.64 | 361553  276847  353075 | 1.28  1.01  1.33 |
| Unknown | 1  2  3 | 8954055  9954041  10606949 | 35.35  36.68  40.92 | 11482874  9947947  10654354 | 44.4  36.59  41.21 | 7732325  9463007  10056642 | 29.50  34.30  38.06 | 4181009  4253603  45180646 | 14.81  15.48  17.10 |

**Table S3. Primer sequences for qRT-PCR validation of miRNAs.**

| miRNA | Mature sequence |
| --- | --- |
| mhp-miR10996a | ACACCATCGCATCTCATGTTCC |
| mhp-miR10996b | TCACCATTGCATCTCATGTTCC |
| mhp-miR11011b | TAAGTTCATCCAAACACCATA |
| mhp-miR159c | GAATTCCTTCTCCTCTCCTTT |
| mhp-miR160a | TGCCTGGCTCCCTGTATGCCA |
| mhp-miR169k | TAGCCAAGGATGACTTGCCTG |
| mhp-miR399a | TGCCAAAGGAGAATTGCCCTG |
| mhp-miR477a | ACTCTCCCTCAAGAGCTTCTC |
| mhp-miR827 | TTAGATGACCATCAACGAACA |
| mhp-miR156p | CTGACAGAAGATAGAGAGCAC |
| mhp-miR395d | GTTCCCTTGACCACTTCATTG |
| mhp-miR482 | TTCCCAAGCCCGCCCATTCCTA |
| mhp-miR535b | TGACAAGGAGAGAGAGCACGC |
| mhp-miR7126-5p | AAAGTATCAAGGAGCGCAAAG |
| mhp-miR408a | ATGCACTGCCTCTTCCCTGGC |

**Table S4. Primer sequences for qRT-PCR validation of miRNA target genes.**

| Target name | Target ID | sense | antisense |
| --- | --- | --- | --- |
| ARF17 | XM_008340583.2 | CACCAGATAGCCAGAGTAG | ACATCATCAGAACCACCTT |
| BBP | XM_008341069.2 | CACTGCCAATCTGGAATG | TTATTATTAGGTAGGTAGGAAGGT |
| BRI1 | XM_008355798.2 | TGTCTACAGTTATGGCGTTA | CTCGGTGTCCAGTATCTC |
| DELLA | XM_008394742.1 | TGATGGTGAAGTCCTTGTT | CATTGTGGCTTGAGTCTTG |
| HSP70 | XM_008386051.1 | AGGGAGAGGAGAAGAAGT | TGAGAGTCGTTGAAGTAGG |
| NF-YA | XM_017323460.1 | ATGCTGTAATCTCGTAGGT | ACAATCACATAACATCAAGGT |
| SPL | NM_001294072.1 | CACTTCTTCTTCTCCACATAC | GCCAGATACATCTCACATTAG |
| SPX | XM_017325912.1 | TCTTGCTGGCTTACTTCA | TACTGCTGTTCCGCTATT |
| TMV-RP | XM_017333719.1 | ACTAGAGGTTGTGGATATGG | TCATCAGCATCAGCAAGA |

**Table S5. Mature sequences of identified known miRNAs in *M. hupehensis* root.**

| Family | miRNA ID | Mature sequence |
| --- | --- | --- |
| MIR1511 | mhp-miR1511 | ACCTAGCTCTGATACCATGAA |
| MIR156 | mhp-miR156a-o | TGACAGAAGAGAGTGAGCAC |
|  | mhp-miR156ab/ac | TTGACAGAAGATAGAGAGCAC |
|  | mhp-miR156p-s | CTGACAGAAGATAGAGAGCAC |
|  | mhp-miR156t-w | TTGACAGAAGAGAGAGAGCAC |
|  | mhp-miR156x-z/aa | TGACAGAAGATAGAGAGCAC |
| MIR159 | mhp-miR159a/b | CTTGGATTGAAGGGAGCTCC |
|  | mhp-miR159c | GAATTCCTTCTCCTCTCCTTT |
|  | mhp-miR319a/b-3p | TTGGACTGAAGGGAGCTCCCT |
|  | mhp-miR319c-3p | ATCCAACGAAGCAGGAGCTGA |
|  | mhp-miR319c-5p/h | GAGCTCTTCTTCAGTCCAGTCC |
| MIR160 | mhp-miR160-e | TGCCTGGCTCCCTGTATGCCA |
| MIR162_1 | mhp-miR162a/b | TCGATAAACCTCTGCATCCAG |
| MIR164 | mhp-miR164a | TGGAGAAGCAGGGCACATGCC |
|  | mhp-miR164b-f | TGGAGAAGCAGGGCACGTGCA |
| MIR167_1 | mhp-miR167a | AGATCATCTGGCAGTTTCACC |
|  | mhp-miR167b-g | TGAAGCTGCCAGCATGATCTA |
|  | mhp-miR167h-j | TGAAGCTGCCAGCATGATCTTA |
| MIR168 | mhp-miR168a/b | TCGCTTGGTGCAGGTCGGGAA |
| MIR169_1 | mhp-miR169b | TAGCCAAGGATGATTTGCCTGC |
|  | mhp-miR169c/d | TAGCCAAGGATGACTTGCCCG |
|  | mhp-miR169e/f | TGAAGAGAAGAGCGTTGTTTGG |
| MIR171_1 | mhp-miR171c-e | TGATTGAGCCGCGCCAATATC |
|  | mhp-miR171f-3p | TTGAGCCGTGCCAATATCACG |
|  | mhp-miR171g/h | TGATTGAGCCGTGCCAATATC |
|  | mhp-miR171j-l | TTGAGCCGCGCCAATATCACT |
|  | mhp-miR171m/n | TTGAGCCGTGCCAATATCACA |
|  | mhp-miR171o | TGGGATGTTGGTATGGTTCAA |
| MIR171_2 | mhp-miR171i | TGAGCCGAACCAATATCACTC |
| MIR172 | mhp-miR172a-c | AGAATCTTGATGATGCTGCA |
|  | mhp-miR172d-h/p | AGAATCTTGATGATGCTGCAT |
|  | mhp-miR172m-o | AGAATCTTGATGATGCTGCAG |
| MIR2111 | mhp-miR2111a/b | TAATCTGCATCCTGAGGTTTA |
| MIR2118_2 | mhp-miR2118a-c | CTACCGATGCCACTAAGTCCCA |
| MIR3627 | mhp-miR3627a-c | TCGCAGGAGAGATGGCACTA |
| MIR390 | mhp-miR390a-f | AAGCTCAGGAGGGATAGCGCC |
| MIR393 | mhp-miR393a-c | TCCAAAGGGATCGCATTGATCT |
|  | mhp-miR393d-f | ATCATGCGATCCCTTCGGACG |
| MIR394 | mhp-miR394a/b | TTGGCATTCTGTCCACCTCC |
| MIR395 | mhp-miR395a-c/e/f/h/d-3p/g-3p/i-3p | CTGAAGTGTTTGGGGGAACTC |
|  | mhp-miR395d-5p/g-5p/i-5p/j | GTTCCCTTGACCACTTCATTG |
| MIR396 | mhp-miR396a | TTCCACAGCTTTCTTGAACAG |
|  | mhp-miR396b | TTCCACAGCTTTCTTGAACTG |
|  | mhp-miR396c-e | TTCCACAGCTTTCTTGAACTT |
|  | mhp-miR396f/g | TTCCACGGCTTTCTTGAACTG |
| MIR397 | mhp-miR397a/b | TTGAGTGCAGCGTTGATGAAA |
| MIR398 | mhp-miR398a | TGTGTTCTCAGGTCACCCCTT |
|  | mhp-miR398b/c | TGTGTTCTCAGGTCGCCCCTG |
| MIR399 | mhp-miR399a-c | TGCCAAAGGAGAATTGCCCTG |
|  | mhp-miR399d | TGCCAAAGGAGAGTTGCCCTA |
|  | mhp-miR399e-h | TGCCAAAGGAGATTTGCTCGG |
|  | mhp-miR399i/j | TGCCAAAGGAGAGTTGCCCTG |
| MIR403 | mhp-miR403a/b | TTAGATTCACGCACAAACTCG |
| MIR408 | mhp-miR408a | ATGCACTGCCTCTTCCCTGGC |
|  | mhp-miR408b-d | ACAGGGAAGAGGTAGAGCATG |
| MIR477 | mhp-miR477b | ACTCTCCCTCAAGGGCTTCGAC |
| MIR482 | mhp-miR482a-3p | TTCCCAAGCCCGCCCATTCCTA |
|  | mhp-miR482a-5p | AGGAATGGGCTGTTTGGGAAGA |
|  | mhp-miR482b | TCTTTCCTATCCCTCCCATTCC |
|  | mhp-miR482c | TCTTTCCTAACCCTCCCATTCC |
|  | mhp-miR482d | AATGGAAGGGTAGGAAAGAAG |
| MIR5225 | mhp-miR5225a/b | TCTGTCGAAGGTGAGATGGTGC |
|  | mhp-miR5225c | TCTGTCGTGGGTGAGATGGTGC |
| MIR535 | mhp-miR535a | TGACAACGAGAGAGAGCACGC |
|  | mhp-miR535b/c | TGACAAGGAGAGAGAGCACGC |
|  | mhp-miR535d | TGACGACGAGAGAGAGCACGC |
| MIR7120 | mhp-miR7120a-3p/b-3p | CAGTCTGACAATATAACGTGC |
|  | mhp-miR7120a-5p/b-5p | TGTTATATTGTCAGATTGTCA |
| MIR7121 | mhp-miR7121a-c | TCCTCTTGGTGATCGCCCTGT |
|  | mhp-miR7121d-h | TCCTCTTGGTGATCGCCCTGC |
| MIR1509 | mhp-miR7122a/b | TTATACAGAGAAATCACGGTCG |
| MIR7123 | mhp-miR7123a/b | AAGAGCGGGATGTGTAAAAGG |
| MIR7124 | mhp-miR7124a/b | CACCAATATCAACTTTATTTG |
| MIR7125 | mhp-miR7125 | CGAACTTATTGCAACTAGCTT |
| MIR7127 | mhp-miR7127a/b | ATACTCATCGAATTTGTCATA |
| MIR827_4 | mhp-miR827 | TTAGATGACCATCAACGAACA |
| Undefined | mdm-miR10979 | CTTGCCGATAGATTTGGGGAG |
|  | mdm-miR10980a/b | CACCTGGGACTTGCAGCCATG |
|  | mdm-miR10982a-d | CGGAATGAAGCTTACGAGAATG |
|  | mdm-miR10984a-5p/4b-5p | GGTAATTGACTGTGAAATCGT |
|  | mdm-miR10984b-3p | CTCACGTACGCTGTCCCGAGAA |
|  | mdm-miR10985 | CCACTCGTAGTGAAACAGTTG |
|  | mdm-miR10986 | TGGCACCAAAGTCACCACCCG |
|  | mdm-miR10991a-e | CGAGCCATTGAAATTCGATCC |
|  | mdm-miR10993c-f | ACATGTGGTGTACCATCCTGT |
|  | mdm-miR10995 | CAAGCTTCCTCTTCATACTCGT |
|  | mdm-miR10996a | ACACCATCGCATCTCATGTTCC |
|  | mdm-miR10996b | TCACCATTGCATCTCATGTTCC |
|  | mdm-miR10998 | CTTGGGATTCAGTCTAGGACTT |
|  | mdm-miR11000 | GTGTTCCAAAGAAATCCGGAGT |
|  | mdm-miR11002c-5p | GAGGATGAGCTTCGGCGGTGA |
|  | mdm-miR11004 | GTATTCTTTCATCTTCTACTA |
|  | mdm-miR11006 | CAATGGGGAGGAGTCATTCGTA |
|  | mdm-miR11008 | GTGACCGCACAAAATAGAAGA |
|  | mdm-miR11011a | AAGTTCATTCAAACACCATGT |
|  | mdm-miR11011b | TAAGTTCATCCAAACACCATA |
|  | mdm-miR11020 | GACATTACAACGGTTACACGG |
|  | mdm-miR159d-f | TTTGGATTGAAGGGAGCTCTA |
|  | mdm-miR166a-j | TCGGACCAGGCTTCATTCCCC |
|  | mdm-miR169a/g-j | CAGCCAAGGATGACTTGCCGG |
|  | mdm-miR169k-n | TAGCCAAGGATGACTTGCCTG |
|  | mdm-miR169o | TAGCCAGGGATGACTTGCCT |
|  | mdm-miR171a/b/p | TTGAGCCGCGTCAATATCTCC |
|  | mdm-miR319b-5p-g | GAGCTTTCTTCAGTCCACTC |
|  | mdm-miR319d | AACTGCCGACTCATTCACTCA |
|  | mdm-miR3627d | TCCATCCTCCTGTGACATGAA |
|  | mdm-miR391 | TACGCAGGAGAGATGACGCCG |
|  | mdm-miR393g/h | ATCATGCTATCCCTTTGGATT |
|  | mdm-miR395l | CTGAAGTGTTTGGGGGAACCC |
|  | mdm-miR399k | TGCCAAAGGAGAGTTGCCCTT |
|  | mdm-miR477a | ACTCTCCCTCAAGAGCTTCTC |
|  | mdm-miR530a-c | TGCATTTGCACCTGCACTTGT |
|  | mdm-miR7126-3p | TTGCGTTCCACTGATTCTTTCG |
|  | mdm-miR7126-5p | AAAGTATCAAGGAGCGCAAAG |
|  | mdm-miR858 | TTCGTTGTCTGTTCGACCTGA |

**Table S6. Mature sequences of identified novel miRNAs in *M. hupehensis* root.**

| miRNA id | Mature sequence |
| --- | --- |
| novel_mir10 | GGGCCGGTGATGACTATGTTAA |
| novel_mir11 | TGTCGATACGCCCGAGGAGACGCC |
| novel_mir12 | TCATGATCTTGGCCAGCTCCCTCAG |
| novel_mir15 | AGGCCGGTGATGTAGATTGG |
| novel_mir18 | GTTGTAGTATAGTGGTAAGTATT |
| novel_mir21 | CAACATCTTATGTTTCCGCTT |
| novel_mir23 | GAGAAAGAGTTTGAGGAAGTTATT |
| novel_mir25 | GAGCGTGATTAACAGCCTTGTGAC |
| novel_mir26 | TCTCAATGAGGAAGAATGCCCAAAGTGATA |
| novel_mir27 | CCTTTACTATAGCTTCGCAGTGACAACCTT |
| novel_mir29 | CACACTATGGGAGCTGGCCATGCCCGAAGT |
| novel_mir30 | AGAAGTGATGATTTGTACCCA |
| novel_mir32 | TTAGGAATGGGCTGTTTGGGAAGA |
| novel_mir38 | CACTTCCCTTACTTGCCCCC |
| novel_mir43 | GGTGGTCACACGGTTGTCT |

**Table S7. Target genes for identified novel miRNAs in *M. hupehensis* root.**

| miRNA id | Target ID | Description |
| --- | --- | --- |
| novel_mir12 | XM_008367259.2 | TMV resistance protein N-like |
|  | XM_017329948.1 | serine/threonine-protein kinase/endoribonuclease IRE1a-like |
|  | XM_017330564.1 | TMV resistance protein N-like |
|  | XM_017330565.1 | TMV resistance protein N-like |
|  | XM_017334398.1 | TMV resistance protein N-like |
| novel_mir15 | XM_017326037.1 | ribosomal RNA-processing protein 17 |
|  | XM_017326680.1 | LOW QUALITY PROTEIN: HEAT repeat-containing protein 5B-like |
| novel_mir38 | XM_008350718.1 | DNA-directed RNA polymerase V subunit 1-like |
| novel_mir43 | XM_008346070.2 | magnesium transporter MRS2-3-like |
|  | XM_008348078.2 | magnesium transporter MRS2-3-like |
|  | XM_008377031.1 | micronuclear linker histone polyprotein-like |
|  | XM_008392360.1 | 4-hydroxyphenylpyruvate dioxygenase-like |
|  | XM_017324637.1 | magnesium transporter MRS2-3-like |
|  |  |  |

**Table S8. Target genes for identified known miRNAs in H_2_S alleviates alkaline salt and alkaline salt stress in *M. hupehensis* root.**

| **miRNA name** | **Target ID** | **Description** | **miRNA name** | **Target ID** | **description** |
| --- | --- | --- | --- | --- | --- |
| **Control-H_2_S+AS** |  |  | **Control-AS** |  |  |
| mhp-miR160a-e | XM_008340583.2 | auxin response factor 17 | mhp-miR156p-s | XM_017324348.1 | squamosa promoter-binding-like protein X1 |
|  | XM_008368452.2 | auxin response factor 18-like isoform X2 |  | NM_001294082.1 | squamosa promoter-binding-like protein 13A |
|  | XM_008368451.2 | auxin response factor 18-like isoform X1 |  | XM_017337264.1 | squamosa promoter-binding-like protein 13A isoform X1 |
|  | XM_008387102.2 | auxin response factor 18 |  | XM_008394575.2 | squamosa promoter-binding-like protein 13A isoform X1 |
|  | XM_008357399.2 | auxin response factor 18-like isoform X2 |  | XM_017336994.1 | squamosa promoter-binding-like protein 2 |
|  | XM_017328042.1 | auxin response factor 18-like |  | XM_017324349.1 | squamosa promoter-binding-like protein 19 isoform X1 |
|  | XM_008357398.2 | auxin response factor 18-like isoform X1 |  | XM_017323697.1 | squamosa promoter-binding-like protein 2 |
|  | XM_008349257.2 | auxin response factor 18-like |  | NM_001294072.1 | squamosa promoter-binding-like protein 18 |
|  | XM_008387101.2 | auxin response factor 18 |  | XM_008391666.2 | squamosa promoter-binding-like protein 2 |
|  | XM_008347179.2 | auxin response factor 18-like |  | XM_008391170.2 | squamosa promoter-binding-like protein 6 |
|  | XM_008354469.2 | auxin response factor 17-like |  | XM_008376003.2 | squamosa promoter-binding-like protein 13A |
|  | XM_008389372.2 | auxin response factor 18-like isoform X2 |  | XM_008361863.2 | squamosa promoter-binding-like protein 6 |
|  | XM_008361074.2 | auxin response factor 18-like |  | XM_008393866.2 | squamosa promoter-binding-like protein 9 |
|  | NM_001294068.1 | auxin response factor 18-like |  | XM_017323790.1 | squamosa promoter-binding-like protein 6 |
|  | XM_008349550.2 | auxin response factor 17-like |  | XM_008342005.2 | squamosa promoter-binding-like protein 2 |
|  | XM_017328045.1 | auxin response factor 18-like |  | XM_008342016.2 | squamosa promoter-binding-like protein 19 |
| mhp-miR399a-e | XM_008385516.2 | ubiquitin-conjugating enzyme E2 24 |  | XM_008376434.2 | squamosa promoter-binding-like protein 6 |
|  | XM_008373144.2 | ubiquitin-conjugating enzyme E2 24 |  | XM_008377572.2 | squamosa promoter-binding-like protein 7 isoform X1 |
| mhp-miR11011b | XM_017333719.1 | TMV resistance protein N-like |  | XM_017324350.1 | squamosa promoter-binding-like protein 19 isoform X1 |
|  | XM_017327993.1 | TMV resistance protein N-like |  | XM_008391665.2 | squamosa promoter-binding-like protein 2 |
|  | XM_017334031.1 | TMV resistance protein N-like |  | XM_008348563.2 | squamosa promoter-binding-like protein 6 |
|  | XM_017332278.1 | TMV resistance protein N-like |  | XM_008377574.2 | squamosa promoter-binding-like protein 7 isoform X2 |
|  | XM_017330692.1 | TMV resistance protein N-like |  | XM_008342004.2 | squamosa promoter-binding-like protein 2 |
|  | XM_017325608.1 | TMV resistance protein N-like |  | XM_008343068.2 | squamosa promoter-binding-like protein 19 isoform X1 |
|  | XM_008383948.2 | TMV resistance protein N-like |  | XM_008394574.2 | squamosa promoter-binding-like protein 13A isoform X1 |
|  | XM_008375054.2 | TMV resistance protein N-like |  | XM_008394573.2 | squamosa promoter-binding-like protein 13A isoform X1 |
|  | XM_008359119.2 | TMV resistance protein N-like |  | XM_017323699.1 | squamosa promoter-binding-like protein 2 |
|  | XM_017334060.1 | TMV resistance protein N-like | mhp-miR390a-f | XM_008355798.2 | receptor-like protein kinase BRI1-like 3 |
|  | XM_017331309.1 | TMV resistance protein N-like |  | XM_008355790.2 | receptor-like protein kinase BRI1-like 3 |
|  | XM_017325231.1 | TMV resistance protein N-like |  | XM_008355783.2 | receptor-like protein kinase BRI1-like 3 |
|  | XM_017323993.1 | TMV resistance protein N-like |  | XM_017335006.1 | leucine-rich repeat receptor-like serine/threonine-protein kinase At3g14840 |
|  | XM_017329453.1 | TMV resistance protein N-like | mhp-miR394a/b | XM_008342892.2 | aluminum-activated malate transporter 12-like |
|  | XM_017326291.1 | TMV resistance protein N-like |  | XM_008391663.2 | F-box only protein 6 |
|  | XM_017333286.1 | TMV resistance protein N-like |  | XM_008354826.2 | Fbox protein |
|  | XM_017325206.1 | TMV resistance protein N-like | mhp-miR160a-e | XM_008340583.2 | auxin response factor 17 |
|  | XM_008348041.2 | TMV resistance protein N-like | mhp-miR11011b | XM_017333719.1 | TMV resistance protein N-like |
|  | XM_008346194.2 | TMV resistance protein N-like | **H_2_S-AS** |  |  |
|  | XM_008361255.2 | TMV resistance protein N-like | mhp-miR319a/b-3p | XM_008344646.2 | transcription factor TCP4-like |
|  | XM_017333284.1 | TMV resistance protein N-like | mhp-miR160a-e | XM_008340583.2 | auxin response factor 17 |
|  | XM_017328695.1 | WRKY transcription factor 52 | mhp-miR394a/b | XM_008342892.2 | aluminum-activated malate transporter 12-like |
|  | XM_008343830.2 | LOW QUALITY PROTEIN: auxin transport protein BIG-like | mhp-miR827 | XM_017325912.1 | SPX domain-containing membrane protein At4g22990-like |
|  | XM_008369909.2 | TMV resistance protein N-like | **Control-H_2_S** |  |  |
|  | XM_017325208.1 | TMV resistance protein N-like | mhp-miR408a | XM_008341069.2 | basic blue protein-like |
|  | XM_017331306.1 | TMV resistance protein N-like | mhp-miR408b-d | NR_120968.1 | probable serine/threonine-protein kinase |
|  | XM_008366861.2 | TMV resistance protein N-like |  | XM_017334013.1 | probable serine/threonine-protein kinase |
|  | XM_017331307.1 | TMV resistance protein N-like |  | XM_008380690.2 | probable serine/threonine-protein kinase |
|  | XM_008350560.2 | TMV resistance protein N-like |  | XM_008367106.2 | probable serine/threonine-protein kinase |
|  | XM_008363066.1 | TMV resistance protein N-like |  | XM_008341251.2 | probable serine/threonine-protein kinase |
|  | XM_017325213.1 | TMV resistance protein N-like | mhp-miR827 | XM_017325912.1 | SPX domain-containing membrane protein At4g22990-like |
|  | XM_017332390.1 | TMV resistance protein N-like |  | XM_008348407.2 | SPX domain-containing membrane protein At4g22990-like |
|  | XM_008348042.2 | TMV resistance protein N-like |  |  |  |
|  | XM_008348043.2 | TMV resistance protein N-like | mhp-miR169a/g-j | XM_008369843.2 | nuclear transcription factor Y subunit A-1-like isoform X1 |
|  | XM_008360206.2 | TMV resistance protein N-like |  | XM_008362229.2 | nuclear transcription factor Y subunit A-3-like |
|  | XM_017327505.1 | TMV resistance protein N-like |  | XM_008362229.2 | nuclear transcription factor Y subunit A-3-like isoform X1 |
|  | XM_008379654.1 | TMV resistance protein N-like |  | XM_008362228.2 | nuclear transcription factor Y subunit A-3-like isoform X1 |
|  | XM_017331308.1 | TMV resistance protein N-like |  | XM_017323460.1 | nuclear transcription factor Y subunit A-5-like |
|  | XM_008382638.2 | TMV resistance protein N-like |  | XM_008346459.2 | nuclear transcription factor Y subunit A-1-like |
|  | XM_017325205.1 | protein SUPPRESSOR OF npr1-1, CONSTITUTIVE 1-like |  | XM_008346217.2 | nuclear transcription factor Y subunit A-9-like |
|  | XM_017331305.1 | TMV resistance protein N-like |  | XM_008362227.2 | nuclear transcription factor Y subunit A-3-like isoform X1 |
|  | XM_008342506.2 | TMV resistance protein N-like |  |  |  |
|  | XM_008384257.2 | putative disease resistance protein At4g11170 |  |  |  |
|  | XM_017333727.1 | TMV resistance protein N-like |  |  |  |
|  | XM_017332455.1 | TMV resistance protein N-like |  |  |  |
|  | XM_017325207.1 | TMV resistance protein N-like |  |  |  |
|  | XM_008363043.2 | TMV resistance protein N-like |  |  |  |
|  | XM_017325209.1 | TMV resistance protein N-like |  |  |  |
|  | XM_017323994.1 | TMV resistance protein N-like |  |  |  |
|  | XM_017332248.1 | TMV resistance protein N-like |  |  |  |
|  | XM_008348040.2 | TMV resistance protein N-like |  |  |  |
| mhp-miR827 | XM_017325912.1 | SPX domain-containing membrane protein At4g22990-like |  |  |  |
|  | XM_008348407.2 | SPX domain-containing membrane protein At4g22990-like |  |  |  |
| mhp-miR159c | XM_008388508.2 |  |  |  |  |
|  | XM_008387451.2 | MADS-box protein JOINTLESS |  |  |  |
|  | XM_008365515.2 | MADS-box protein JOINTLESS-like isoform X1 |  |  |  |
|  | XM_008368432.2 | indole-3-pyruvate monooxygenase YUCCA4 isoform X1 |  |  |  |
|  | XM_008356757.2 | clathrin heavy chain 2-like |  |  |  |
|  | NM_001293986.1 | MADS-box protein JOINTLESS |  |  |  |
|  | XM_008386051.1 | heat shock 70 kDa protein |  |  |  |
|  | XM_008386038.2 | heat shock 70 kDa protein |  |  |  |
|  | XM_008360988.2 | MADS-box protein JOINTLESS-like |  |  |  |
|  | XM_008365516.2 | MADS-box protein JOINTLESS-like isoform X1 |  |  |  |
|  | XM_008365518.2 | MADS-box protein JOINTLESS-like isoform X2 |  |  |  |
|  | XM_008349751.1 | heat shock cognate 70 kDa protein-like |  |  |  |
|  | XM_008386045.1 | heat shock 70 kDa protein |  |  |  |
|  | XM_017330136.1 | dormancy-associated MADS-box transcription factor |  |  |  |
|  | XM_008365517.2 | MADS-box protein JOINTLESS-like isoform X1 |  |  |  |
| mhp-miR169k-n | XM_017326310.1 | nuclear transcription factor Y subunit A-10-like |  |  |  |
|  | XM_008362229.2 | nuclear transcription factor Y subunit A-3-like isoform X1 |  |  |  |
|  | XM_008362228.2 | nuclear transcription factor Y subunit A-3-like isoform X1 |  |  |  |
|  | XM_008388897.2 | nuclear transcription factor Y subunit A-10-like |  |  |  |
|  | XM_008348710.2 | nuclear transcription factor Y subunit A-4-like |  |  |  |
|  | XM_008362227.2 | nuclear transcription factor Y subunit A-3-like isoform X1 |  |  |  |
|  | XM_008345809.2 | nuclear transcription factor Y subunit A-3-like |  |  |  |
|  | XM_017323460.1 | nuclear transcription factor Y subunit A-5-like |  |  |  |
|  | XM_008377041.2 | nuclear transcription factor Y subunit A-4-like |  |  |  |
|  | XM_017326316.1 | nuclear transcription factor Y subunit A-10-like |  |  |  |
